# A Cross‐Sectional Study of the Quality of Online Information on Periodontal Surgery

**DOI:** 10.1002/cre2.70195

**Published:** 2025-08-05

**Authors:** William Weng Nian Mak, Sushil Kaur, Maurice J. Meade

**Affiliations:** ^1^ Dept. of Periodontics Adelaide Dental School University of Adelaide Adelaide Australia; ^2^ Orthodontic Unit Adelaide Dental School University of Adelaide Adelaide Australia

**Keywords:** health literacy, online information, periodontal surgery, quality of information, website readability

## Abstract

**Objective:**

To investigate the quality of online information provided by dental‐related websites regarding periodontal surgery.

**Methods:**

The term “Gum Surgery” was entered into three search engines (Google, Yahoo, and Bing). The content of websites satisfying selection criteria was assessed with five validated quality of information tools (DISCERN, The Patient Education Materials Assessment Tool [PEMAT], Journal of the American Medical Association [JAMA] benchmarks, and HONCode and @TRUST certification). The Simple Measure of Gobbledygook (SMOG) was used to evaluate the readability of content.

**Results:**

A total of 55 websites satisfied selection criteria. The mean (SD) DISCERN score for all website categories was 2.89 (0.57). The quality of information related to the risks of each treatment scored poorly in most websites. The healthcare portals obtained the highest mean PEMAT score of 71.74%, a statistically significant outcome. Healthcare portal websites also recorded the highest mean (SD) JAMA score of 3.72 (0.75) out of 4. The mean (SD) SMOG score was 9.56 (1.07). Cohen's *κ* inter‐rater reliability for DISCERN and PEMAT scores were 0.75 and 0.79, respectively.

**Conclusion:**

The information available online about periodontal surgery was variable and difficult to read, falling short of established standards for accuracy, reliability, and credibility. Vital information was often omitted.

## 1 Introduction

In the Global Oral Health Status Report (GOHSR) published by the World Health Organization (WHO) in 2022, it was reported that 3.5 billion individuals are affected by some form of oral disease. Among the most prevalent noncommunicable oral pathologies, periodontal disease ranks just below dental caries, affecting over 1 billion people globally (World Health Organisation 2022). Periodontal disease can lead to periodontal recession, mobility of teeth, and tooth loss. This frequently results in poorer quality of life due to difficulties with eating and speaking (World Health Organisation 2022). In addition, periodontitis has an economic impact with an estimated $54 billion in direct treatment costs and $25 billion in indirect costs recorded globally in 2017 (Sanz et al. 2020). Consequently, it is important to treat periodontal disease promptly. The demand for periodontal surgical services appears to be increasing (Fact. MR 2023), as treatment of periodontal disease commonly involves interventions such as access flap surgery, resective flap surgery, and regenerative surgery (Sanz et al. 2022). Additional required periodontal surgical procedures include the exposure of ectopic teeth to aid orthodontic traction and alignment, dental implant surgery, crown lengthening surgery, and frenectomy (Wennström and Zucchelli 2015).

With the advancement of technology over the past 30 years, the Internet has become an important source of information for health‐related services (Bujnowska‐Fedak et al. 2019). The same timeframe has seen the emergence of several quality of health information tools, such as DISCERN, the Patient Education Materials Assessment Tool (PEMAT), HONCode Certification, and the Journal of the American Association (JAMA) benchmarks (Hanif et al. 2009; Shoemaker et al. 2013). These can be used to help healthcare providers and/or patients determine the quality of written, and in some cases, audio‐visual health information (Charnock et al. 1999; Shoemaker et al. 2013; Silberg 1997).

Accurate and reliable information is essential for patients to validly consent or refuse proposed treatment interventions (Barstow et al. 2018). Health literacy relates to the social and cognitive ability to look for, understand, and apply knowledge to advance health and well‐being (Nutbeam 2000). This includes the ease with which written material related to health can be read. The National Institutes of Health has reported that the recommended readability score for health‐related information should align with the average literacy level of an American adult, which is approximately at an eighth‐grade school level (Rooney et al. 2021). It was reported that during the recent COVID‐19 pandemic, close to 50% of adults in Europe experienced challenges associated with health literacy (Paakkari and Okan 2020). Moreover, an estimated 3 billion individuals globally struggle with fundamental literacy skills in reading and writing (World Literacy Foundation 2018). Consequently, ensuring the ease of comprehension and readability of pertinent online content is essential for effectively communicating health information to the general population.

Several studies have reported on the quality and readability of online content regarding oral conditions and interventions (Meade and Dreyer 2021; Parvez et al. 2019; Pons‐Fuster et al. 2020). Relevant investigations regarding periodontics, however, are limited. The evidence has indicated that the quality of information regarding periodontal conditions is variable (Chestnutt 2002; Kanmaz and Buduneli 2021). Other studies found that online periodontal‐related information often lacked reliability and quality, with key content missing or inadequately addressed (Schwendicke et al. 2017; Ali et al. 2014; Kanmaz and Buduneli 2021). Corresponding research, however, on periodontal surgery is lacking. Therefore, the purpose of this study was to assess the quality of information contained within the websites related to periodontal surgery. The null hypothesis is that the quality of information on the Internet regarding periodontal surgery is of acceptable standard.

## 2 Materials and Methods

Ethical approval was not needed for this study as it involved the assessment of publicly available information only.

### 2.1 Search Strategy

The purpose of the search strategy was to identify websites containing information related to periodontal surgery. With the aid of Google Trends (Menlo Park, California, the United States), the term “Gum Surgery” was identified as the most widely searched phrase associated with periodontal surgery from 2017 to 2022. This term was chosen because it reflects the language that ordinary laypeople are using to search for information online about periodontal surgery. It was found that “gum surgery” was the most frequently searched phrase compared to other terms, such as periodontal surgery, gum graft surgery, and bone graft surgery. Additionally, using the term “gum surgery” ensures the search term is relevant and inclusive of all types of periodontal surgery, rather than limiting it to a specific surgical procedure. The term “gum surgery” was then entered into the three most commonly used search engines globally (Google, Yahoo, and Bing) (Fox and Duggan 2013; Hockman 2023; Purcell et al. 2012). The searches were conducted on a laptop computer on October 1, 2023. Each search was conducted using “Incognito mode” following clearance of the browsing history and switching off the location tracking function.

Websites were included if they were presented in the English language and provided information related to periodontal surgery. These included surgical procedures performed on the gums (gingiva) to address various conditions related to gum health and the supporting structures of the teeth. Website content related to oral and maxillofacial surgery and/or third molar surgery was excluded from the study.

The unique resource locator (URL) of the first 35 websites from each of the searches was recorded.

### 2.2 Classification of Website Categories

The websites were categorised into five main groups based on their affiliation—“Commercial,” “Professional body,” “Specialist practice,” “General dental practice,” and “Healthcare portal” as described by Bizzi et al. (2017).

### 2.3 Qualitative Assessment

All of the included websites were scored by the main author (W.M.) using the following assessment tools.

#### 2.3.1 JAMA Benchmark

The JAMA Benchmark was also selected to assess the quality and reliability of information based on four benchmark criteria (Table 1) (Silberg 1997). Each of the criteria is given a dichotomous score of 0 (criterion not met) or 1 (criterion met). Based on definitions provided in prior publications, websites fulfilling at least 3 JAMA criteria were considered as high‐quality (Meric 2002; Rothrock et al. 2019).

**Table 1:** Definitions of JAMA Benchmark criteria (Silberg 1997).

Criterion	Definition
Authorship	Authors and contributors, their affiliations, and relevant credentials should be provided.
Attribution	References and sources for all content should be listed clearly, and all relevant copyright information noted.
Disclosure	Website “ownership” should be prominently and fully disclosed, as should any sponsorship, advertising, underwriting, commercial funding arrangements or support, or potential conflicts of interest.
Currency	Dates that content was posted and updated should be indicated.

Abbreviation: JAMA, Journal of the American Medical Association.

#### 2.3.2 DISCERN Instrument

The DISCERN instrument comprises a questionnaire structured into three sections (Table 2). Questions 1–8 assess the reliability of the publication and act as an aid in determining whether the source of information regarding treatment options can be trusted (Charnock et al. 1999). Questions 9–15 address the quality of information on the treatment choices. Question 16 provides the opportunity for an overall rating of the publication. Each question is scored on a Likert scale of 1–5 (1 indicates poor quality and/or inaccurate information and 5 indicates excellent comprehensive and evidence‐based information) (Portillo et al. 2021). The cumulative score can be categorised as “Excellent” (scoring between 63 and 80), “Good“ (51–62), “Fair” (39–50), “Poor” (27–38), and “Very Poor” (15–26) (Bukhari et al. 2021).

### 2.4 Understandability and Actionability Assessment

#### 2.4.1 PEMAT

The PEMAT was chosen to evaluate the understandability and actionability of patient education materials (Shoemaker et al. 2013). This tool aims to address the limitations of other health‐related material assessment tools, which mainly assess print materials. The term “Understandability” is defined as the ability to comprehend and explain the essential messages conveyed in patient education materials by individuals with diverse backgrounds and varying degrees of health literacy (Shoemaker et al. 2013). “Actionability” is defined as the capacity to recognize actions that consumers can take, based on the information presented (Shoemaker et al. 2013). The PEMAT is available in two formats. The PEMAT‐P (Prints) is used to assess printed materials and consists of 17 items that measures understandability and 7 items for actionability. The second format, the PEMAT‐A/V (Audio‐visual), evaluates audio‐visual materials and consists of 13 items measuring understandability and 4 items measuring actionability (Shoemaker et al. 2013). Each item is given a score of 0 (“disagree”), 1 (“agree”), or N/A (when deemed not applicable). A percentage score is then calculated. During the development of the PEMAT, Shoemaker et al. introduced a threshold score of 70% or greater, indicating acceptable understandable or actionable information (Shoemaker et al. 2014). In the present study, both formats of the PEMAT tool were utilized.

### 2.5 Assessment of Trustworthiness

#### 2.5.1 HONCode Certification

The HONCode Certification, developed by the Health On the Net Foundation (HON), was chosen to determine the credibility and reliability of information (Massicotte 2015). Websites that meet the HON certification criteria are awarded a unique seal to place on their webpages. The eight criteria are as listed below:

1. Authority (includes author credentials).
2. Complementarity (information complements without substituting patient–doctor relationship).
3. Confidentiality (protects the privacy of web users).
4. Attribution (acknowledges the source and recency of information).
5. Justifiability (provides evidence to support claims relating to benefit).
6. Transparency (provides valid contact details of authors).
7. Financial disclosure (identifies sources of fundings).
8. Advertising (differentiates between promotional material and informative content) (Health‐on‐the‐Net 2020).

#### 2.5.2 @TRUST Certification

The @TRUST certification seal issued by the American Accreditation Commission International (ACCI) was utilised as an indicator of information trustworthiness (AACI 2023). For websites that meet the eight validation criteria (Author and editor identification, Reference bibliography, Physician–patient ethical considerations, Privacy, Transparency, Risks and benefits, Advertising and marketing, and Medical content review), the AACI (2023) issues the @TRUST certification seal for display on the information provider's webpage. The seal verifies the website as a source of reliable information and assures readers of the quality and transparency of the information.

### 2.6 Readability Assessment

The readability of the information provided on the websites was assessed using the Simple Measure of Gobbledygook (SMOG) instrument (Cheng and Dunn 2015). This instrument gauges the complexity of the language used by counting the number of words with three or more syllables in the ten sentences at the beginning, middle, and end. The square root of the final number is then determined and rounded off to nearest 10. The constant value of 3.1291 was also added to give the SMOG grade.

The content of the individual websites was copied and pasted into a readability formula software (https://readabilityformulas.com/free-readability-formula-tests.php). To minimize errors in assessment, the contents were modified by removing of bullet points and unclear punctuations that may have caused confusion. The readability score was calculated by the software and recorded. The score represents the estimated years of education a person needs to comprehend a piece of writing easily. The recommended readability score for health‐related information should be no higher than eighth‐grade level (Rooney et al. 2021).

### 2.7 Error Study

Thirteen websites, constituting 25% of the sample, were randomly chosen to assess intra‐rater and inter‐rater reliability. A periodontist colleague (T.C.) recorded DISCERN and PEMAT scores for these websites in a separate Excel spreadsheet for inter‐rater evaluation on November 15, 2023. The author reassessed the same websites for intra‐rater reliability on March 2, 2024. The number of observed agreements were entered into GraphPad Statistics software (La Jolla, California, the United States). Cohen's *κ* scores were then calculated.

### 2.8 Statistical Analyses

All data were tabulated in Microsoft Excel software (Microsoft Corporation, Washington, the United States) and statistical analyses were determined via the GraphPad Statistics software facility (La Jolla, California, the United States). Descriptive statistics were reported as mean (SD)s or medians (interquartile ranges [IQRs]) and percentages. The Shapiro–Wilk test was performed to evaluate the normality of the data distribution. In accordance with findings from the normality tests, either a one‐way analysis of variance (ANOVA) or Kruskal–Wallis test was used to compare the mean or median DISCERN, PEMAT, and SMOG scores to determine if there were statistically significant differences between the groups. A point‐biserial correlation test was also performed to determine the relationship between JAMA score and HONCode/@TRUST certification.

## 3 Results

### 3.1 Website Distributions

**Figure 1:**
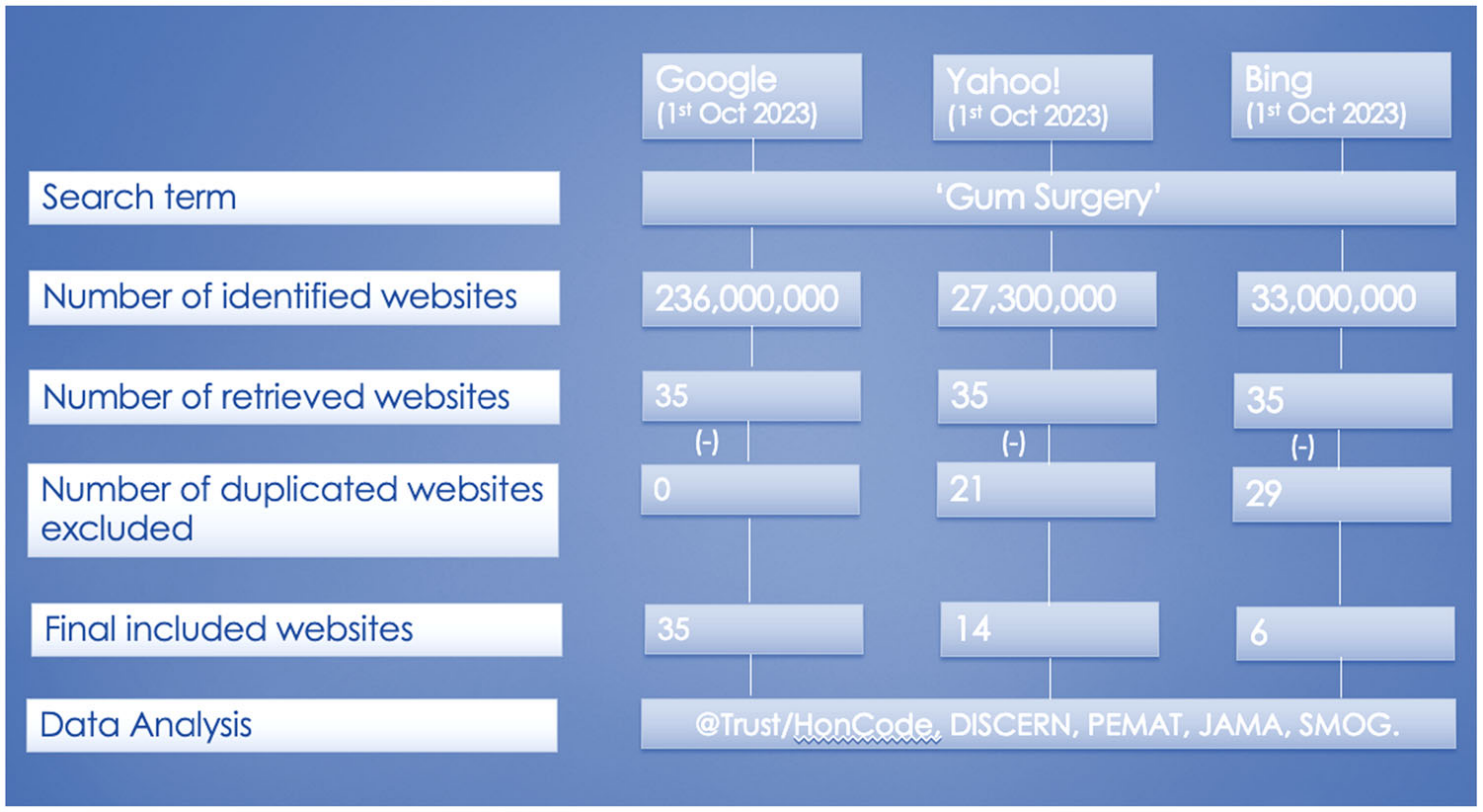
Flowchart of selection process.

**Figure 2:**
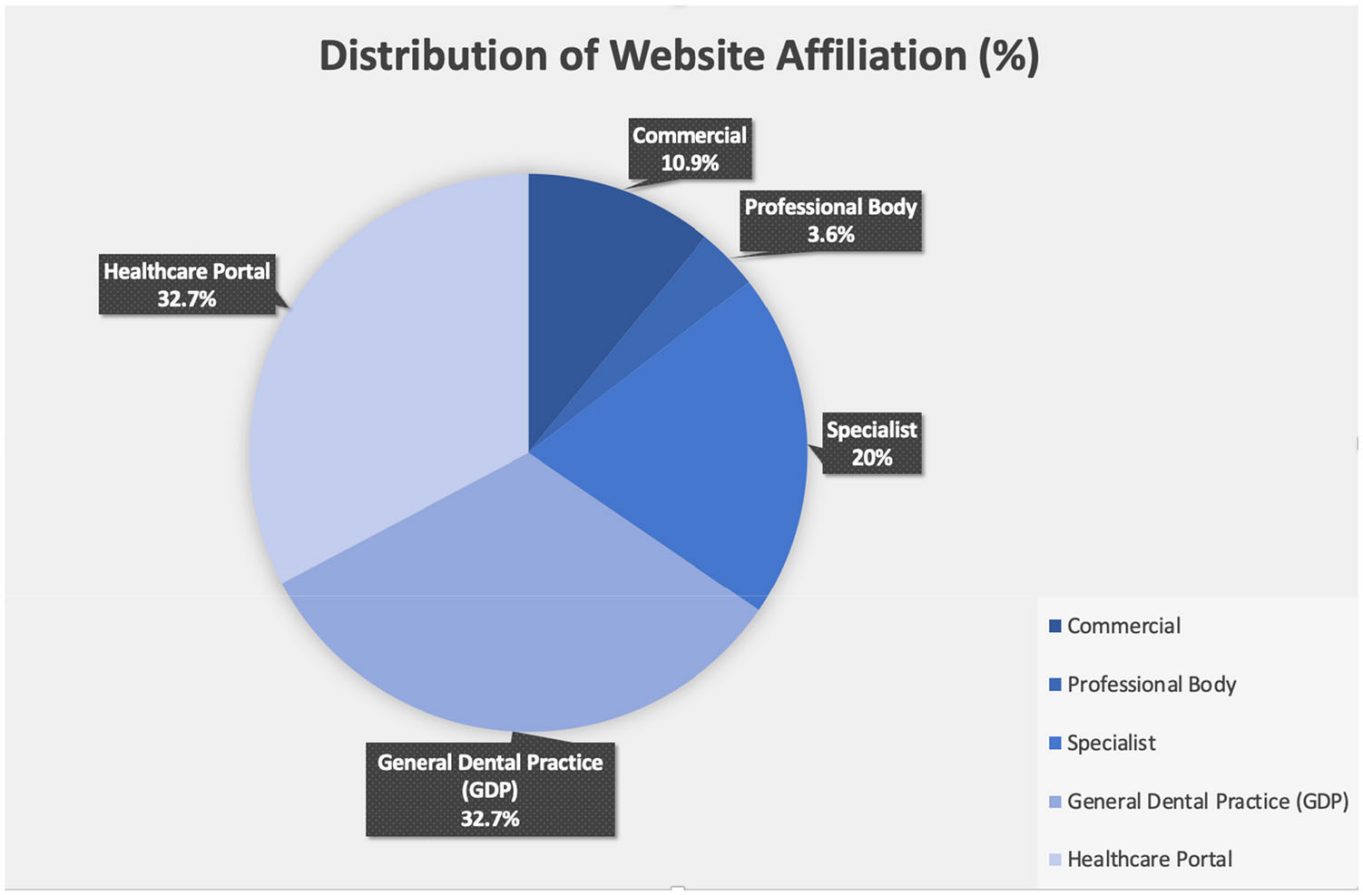
Distribution of website affiliation (*N* = 55). (*N*: Number; %: Percentage).

**Figure 3:**
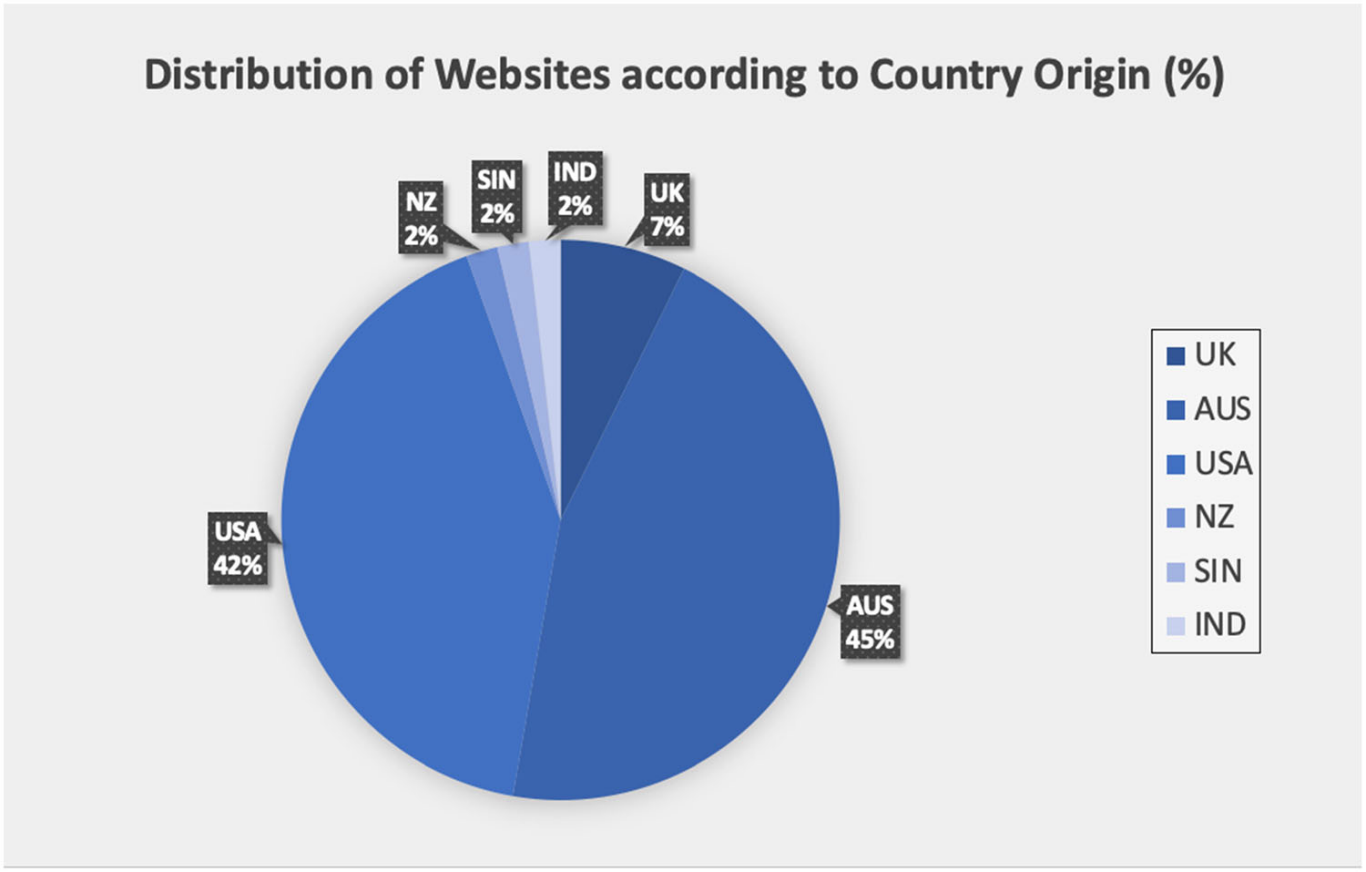
Distribution of websites according to country of origin (*N* = 55). (KEY. UK: United Kingdom; AUS: Australia; USA: United States of America; NZ: New Zealand; SIN: Singapore; IND: India).

A total of 55 websites satisfied selection criteria (Figure 1). Websites authored by general dental practitioners (GDPs) (*N* = 18; 32.7%) and healthcare portals (e.g., Healthline, WebMD, and Mayo Clinic) (*N* = 18; 32.7%) constituted the majority of the included websites (Figure 2). Most websites (*N* = 10; 71.4%) that were obtained through the Yahoo search engine had affiliations with healthcare portals, whereas 74% of the websites retrieved from Google were linked to dental practices (GDPs and Specialists). Figure 3 shows that most of the websites were based in Australia (*N* = 25; 45.5%) and the United States (*N* = 23; 41.8%).

### 3.2 Normality Tests

The Shapiro–Wilk test indicated a normal distribution for the DISCERN scores (*p* = 0.055) and SMOG readability scores (*p* = 0.56), while the PEMAT scores indicated a non‐normal distribution (*p* = 0.017).

### 3.3 DISCERN Score

The mean (SD) scores for each DISCERN item were calculated according to their website categories and an overall mean was determined (Table 2). The mean (SD) DISCERN Score was highest for healthcare portal (3.99 [0.78]) and the cumulative mean score for all categories was 46.24 (1.04). A one‐way ANOVA revealed a significant statistical difference between the different website categories (*p* = 0.0008).

**Table 2:** Mean DISCERN score according to website categories.

DISCERN Items	Mean (SD)					
Section 1:	Commercial *N* = 6 (10.9%)	Professional body *N* = 2 (3.6%)	Specialist *N* = 11 (20%)	GDP *N* = 19 (34.5%)	Healthcare portal *N* = 17 (30.9%)	Overall mean
Is the publication reliable?						
1. Are the aims clear?	4.00 (1.53)	2.00 (1.00)	1.91 (1.56)	2.11 (1.70)	3.78 (1.78)	2.76 (0.93)
2. Does it achieve its aims?	3.80 (1.60)	5.00 (0.00)	4.33 (0.47)	4.17 (1.21)	4.85 (0.53)	4.43 (0.44)
3. Is it relevant?	3.67 (1.49)	5.00 (0.00)	3.64 (1.30)	3.78 (1.23)	4.67 (0.75)	4.15 (0.57)
4. Is it clear what sources of information were used to compile the publication (other than the author or producer)?	1.67 (0.94)	1.00 (0.00)	1.45 (0.78)	1.22 (0.63)	3.78 (1.08)	1.82 (1.00)
5. Is it clear when the information used or reported in the publication was produced?	1.00 (0.00)	1.00 (0.00)	1.00 (0.00)	1.00 (0.00)	4.22 (1.51)	1.64 (1.29)
6. Is it balanced and unbiased?	2.83 (1.57)	4.00 (1.00)	2.18 (1.27)	2.00 (1.11)	4.61 (0.89)	3.12 (1.02)
7. Does it provide details of additional sources of support and information?	2.00 (1.53)	3.00 (0.00)	1.64 (1.23)	1.39 (1.11)	4.17 (0.90)	2.44 (1.02)
8. Does it refer to areas of uncertainty?	1.00 (0.00)	3.00 (2.00)	1.82 (1.53)	1.67 (1.29)	2.83 (1.57)	2.06 (0.75)
Section 2: How good is the quality of information on treatment choices?	Commercial	Professional body	Specialist	GDP	Healthcare portal	Overall mean
9. Does it describe how each treatment works?	3.67 (1.89)	4.00 (1.00)	3.18 (1.64)	4.17 (1.21)	4.72 (0.65)	3.95 (0.51)
10. Does it describe the benefits of each treatment?	3.67 (1.60)	5.00 (0.00)	3.73 (1.54)	3.72 (1.56)	4.78 (0.53)	4.18 (0.58)
11. Does it describe the risks of each treatment?	1.5 (0.5)	1.00 (0.00)	1.91 (1.16)	1.53 (0.70)	3.17 (1.64)	1.82 (0.73)
12. Does it describe what would happen if no treatment were used?	2.5 (1.61)	3.00 (2.00)	2.55 (1.78)	1.94 (1.65)	2.44 (1.77)	2.49 (0.34)
13. Does it describe how the treatment choices affect overall quality of life?	3.33 (1.70)	1.00 (0.00)	2.64 (1.61)	2.39 (1.25)	4.00 (1.45)	2.67 (1.01)
14. Is it clear that there might be more than one possible treatment choice?	4.83 (0.37)	5.00 (0.00)	4.00 (1.54)	3.78 (1.55)	5 (0.00)	4.52 (0.52)
15. Does it provide support for shared decision‐making?	1.17 (0.37)	1.00 (0.00)	1.27 (0.62)	1.61 (1.06)	2.78 (1.81)	1.57 (0.64)
Section 3: What is the overall rating of the publication?	Commercial	Professional body	Specialist	GDP	Healthcare portal	Overall mean
16. Based on the answers to all of the above questions, rate the overall quality of the publication as a source of information about treatment choices	2.33 (1.25)	2.00 (1.00)	2.55 (1.08)	2.11 (1.05)	4.06 (0.85)	2.61 (0.75)
Mean for each affiliation (SD)	2.69 (1.17)	2.88 (1.58)	2.49 (0.99)	2.41 (1.08)	3.99 (0.78)	2.89 (0.57)

### 3.4 PEMAT Score

**Figure 4:**
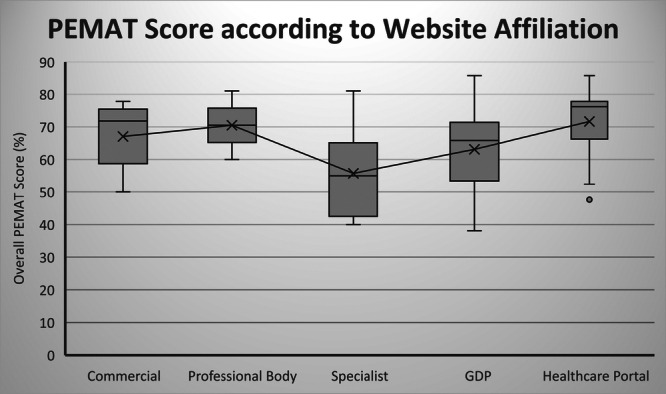
Box plot of PEMAT scores according to website category (Mean, Median, IQR). Mean is marked by ‘X’ on the chart. Key: PEMAT (Patient Education Materials Assessment Tool); GDP (general dental practitioner); IQR (interquartile range).

Figure 4 illustrates the median and IQR for the PEMAT score for each website category. The median PEMAT score for each category was: 71.8% (Commercial), 70.5% (Professional), 55.0% (Specialist), 65.9% (GDP), and 76.2% (Healthcare portal). A Kruskal–Wallis test revealed a significant difference between the different website categories (*p* = 0.046).

### 3.5 Readability Score

**Figure 5:**
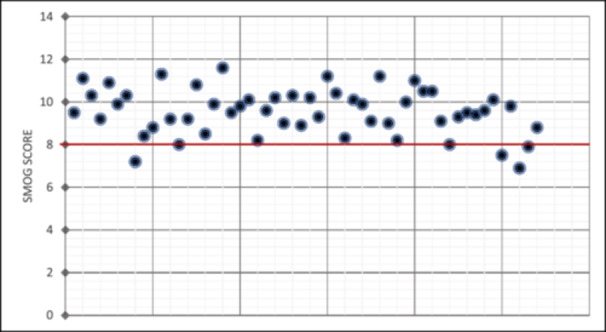
Scatterplot of SMOG score for each website (*n* = 55). † Red line denotes recommended readability score for health‐related information (Rooney et al. 2021).

The SMOG scores for all websites are illustrated on a scatterplot (Figure 5). It can be observed that almost all of the websites scored higher than the recommended readability score for health‐related information, which is up to eighth‐grade level (Rooney et al. 2021). The mean (SD) SMOG score was 9.56 (1.07). ANOVA testing indicated that the difference between the groups was not statistically significant (*p* value = 0.242).

### 3.6 Mean JAMA Score for Individual Category

Twenty‐one websites achieved a JAMA score of 3 and above. Healthcare portals achieved the highest mean score (SD) of 3.72 (0.75) (Table 3).

**Table 3:** Mean JAMA Score according to category.

Website category	Mean JAMA score (SD)
Healthcare portal	3.72 (0.75)
Commercial	1.50 (0.55)
GDP	1.33 (0.77)
Specialist	1.18 (0.6)
Professional	2.00 (1.41)

Abbreviations: GDP, General dental practitioner; JAMA, Journal of the American Medical Association; SD, Standard deviation.

### 3.7 Correlation Between JAMA Score and HONCode/@TRUST Certification

**Figure 6:**
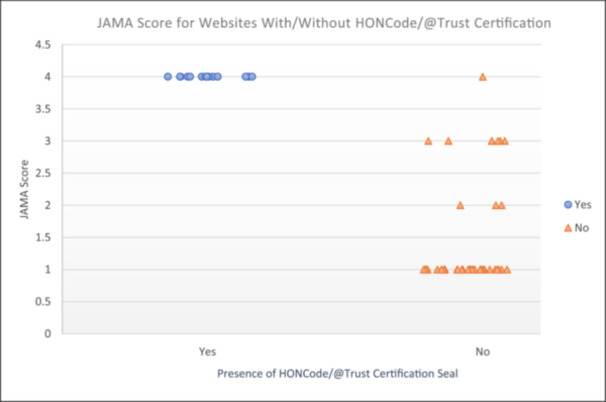
Jitter plot of JAMA score for each website according to the presence or absence of HONCode/@TRUST Certification Seal (*N* = 55). (“Yes” represents websites with certification seal; “No” represents websites without certification seal).

A jitter plot (Figure 6) was charted to demonstrate the association between JAMA score for each website and HONCode/@TRUST certification. A point‐biserial correlation test was also performed to determine the relationship. There was a strong positive correlation between the variables (*r* = 0.828; *p* < 0.001). All websites with HONCode/@TRUST certification had a JAMA score of 4. By contrast, the JAMA score for websites without either the HONCode or @TRUST certification had a wide distribution of scores between 1 and 4. The majority of the websites without certification (*N* = 30; 73.17%) received a JAMA score of 1. Out of the five affiliation categories, “Healthcare portal” was the only group that had HONCode or @TRUST certification, with 77.8% (*N* = 14) of its websites displaying the seal of certification.

### 3.8 Inter‐Rater and Intra‐Rater Reliability

The inter‐rater reliability for DISCERN and PEMAT scores, were 0.75 and 0.79, respectively. Intra‐rater reliability testing yielded values of 0.85 and 0.95.

## 4 Discussion

This study appears to be the first to investigate the quality of online information related to periodontal surgery. The findings revealed considerable variability in the quality of content, with most websites exceeding recommended readability levels, making them difficult for the average user to understand. Given the high prevalence of periodontal disease and the widespread use of the Internet as a health information source, this study's significance cannot be overstated. The ability of patients to access reliable, comprehensible information is essential for making informed decisions about their health.

In examining the distribution of website categories across different search engines, this study found that 74% of the websites retrieved from Yahoo were affiliated with healthcare portals, a distribution not previously analysed in the context of Yahoo search results. In contrast, 74% of the websites retrieved from Google were linked to dental practices, including both GDPs and specialists. These findings align with similar search outcomes reported by Bizzi et al. (2017), suggesting that search engines like Yahoo and Google may favour different types of content, which could influence the accessibility and reliability of health‐related information.

To ensure the comprehensiveness of the search, this study focused on the first 35 websites retrieved from each search engine. Although prior studies have indicated that examining the first 30 results is typically sufficient (Ali et al. 2014), the inclusion of five additional websites ensured a more thorough exploration of available content. This approach was further supported by a 2017 report that found 71%–92% of users only review the first page of search results (10 results per page), with fewer than 6% examining results on the second page (Shelton 2017), highlighting the importance of including the most relevant websites in the analysis.

As periodontal surgery is a nonreversible procedure, it is crucial for patients to have a sound understanding of details regarding treatment interventions to validly consent to treatment (Baxley 2008). Research by Sherlock and Brownie (2014) demonstrated that only 21% of patients could recall the risks and complications discussed during their initial consultation, emphasizing the need for accurate and reliable information from supplementary sources (Sherlock and Brownie 2014). In fact, a 2001 survey revealed that 52 million American adults have used the Internet to search for health‐related information (Diaz et al. 2002). Despite the importance of online health information, this study's findings highlight the generally poor to moderate quality of the websites assessed, as indicated by the DISCERN scores. Most of the websites performed well in presenting pertinent details regarding the benefits of each treatment. However, the information concerning the risks of these treatments was deficient. In addition, information regarding the potential outcomes of not undergoing treatment was inadequate. This is a potential cause for concern, as Internet users may not be aware that much online health information is suboptimal (Ofcom 2021). In addition, many users may not, or have the capacity to, critically evaluate the reliability of information found on the Internet (Ofcom 2021). These findings underscore the need for healthcare providers to guide patients toward trusted and well‐researched resources.

In terms of the DISCERN score, healthcare portal websites had the highest overall mean score of 3.99 (0.78), indicating above‐moderate quality. According to Charnock's 1998 criteria, publications with a score of 3 are considered of ‘moderate’ quality, positioning healthcare portal websites above this threshold. However, the combined mean for all website categories scored less than this, consistent with the scores of 1.84–3.9 recorded by previous studies in the dental domain (Goodrum and Johal 2023; McMorrow and Millett 2016; Meade and Dreyer 2021). It was noted that all categories, apart from healthcare portals, were equally poor in describing treatment risks. Professional body and healthcare portal websites also performed well in offering balanced and unbiased information. This may be due to input from credible experts in the field and adherence to strict publication standards, such as HONCode or @TRUST certification. Additionally, their commitment to serving the public interest over commercial agendas may contribute to the reliability of the information they provide.

The PEMAT instrument, designed to assess the understandability and actionability of health materials, also revealed that healthcare portal websites achieved the highest scores among the website categories. To the authors' knowledge, this appears to be the first study related to periodontology that has evaluated the understandability and actionability of patient education materials using the PEMAT instrument. This is of importance as online information should not only convey knowledge but also empower individuals to take specific actions in response to that information (Shoemaker et al. 2014).

In assessing the overall trustworthiness of the websites, this study utilized the JAMA benchmarks, which are widely used as a quick assessment tool for health information quality (Cassidy and Baker 2016). Besides JAMA, the HONCode and @TRUST certification seals can also be used to determine website trustworthiness. Presently, this study appeared to be among the first to incorporate the @TRUST certification in assessing the quality and credibility of online information. It was found that websites with HONCode or @TRUST certification seals consistently received a JAMA score of 4. This observation was not surprising, given that similar criteria are utilized in the three instruments. In contrast, the majority of websites without HONCode and @TRUST certification received a JAMA score of 1. The HONCode and @TRUST certification were only observed in healthcare portals. These findings corresponded with a 2017 study, which found that health portals were one of the most reliable websites with significantly higher JAMA scores and more likely to have HONCode certification (Bizzi et al. 2017). As such, Internet users can be reasonably confident of the trustworthiness of websites that display HONCode or @TRUST certification seals.

The SMOG readability test, which is regarded as the gold standard for evaluating health information readability (Cheng and Dunn 2015), revealed a mean readability score of 9.56 for the websites in this study. This corresponded with the literacy level expected of a person who has completed 9–10 years of education (typically equivalent to individuals who are 14–16 years old) in public education in the United States. However, the National Institutes of Health have recommended that the reading difficulty for health‐related information should be no more than eighth‐grade level, which is the purported average literacy level of an American adult (Rooney et al. 2021). In the examination of 39 websites pertaining to dental implant surgery, Jayaratne et al. reported that the mean readability level of these websites approximated to grade 11 (Jayaratne et al. 2014). This finding closely corresponds with our current research, emphasizing the challenge of providing easy to read online information about periodontal surgery.

The limitations of this study mirror those of other cross‐sectional surveys in the field. As the Internet is a dynamic medium, a search at a different timepoint may have yielded different websites for evaluation. In addition, the present study was limited to websites in the English language. Therefore, the findings may not be applicable to information in languages other than English. It is crucial to highlight that the assessment instruments such as PEMAT, DISCERN, JAMA, and HONCode are limited to evaluating the reliability, trustworthiness, and actionability of healthcare content, rather than its accuracy. Criteria such as “Attribution,” “Justifiability,” and “Reference bibliography” are important in assessing the credibility of information, but they do not necessarily guarantee factual accuracy. Therefore, patients should exercise caution and seek additional verification from reliable healthcare professionals to ensure the accuracy and completeness of the information before making informed decisions about their health. A recent study adapted a tool that was originally designed for assessing drug advertisements to evaluate the accuracy of statements regarding orthodontic products (Meade et al. 2024). Their study revealed a positive correlation between DISCERN scores and the accuracy‐of‐information scores. This suggests the potential for the combined use of DISCERN and an accuracy‐of‐information instrument to overcome the deficiencies of each tool and provide a more comprehensive evaluation of both reliability and factual accuracy in online health information.

## 5 Conclusion

The information available online about periodontal surgery was variable and generally deemed below average, falling short of established standards for reliability and trustworthiness. However, healthcare portal websites scored high for JAMA and were rated as “Excellent” (Mean: 3.99 ± 0.78; Total: 63.84 ± 0.78) in the DISCERN tool, indicating that this category is a good source of reliable and trustworthy information. In addition, most websites were difficult to read and exceeded recommended literacy levels. Authors of online content should consider the use of quality of information and readability tools to aid in the provision of reliable and trustworthy information for those seeking information on periodontal surgery.

## Author Contributions

William Weng Nian Mak contributed to conceptualization, formal analysis, investigation, methodology, validation, visualization, original draft preparation and editing. Sushil Kaur contributed to project support, administration and supervision. Maurice J. Meade contributed to methodology, visualization, supervision, and manuscript review and editing.

## Ethics Statement

The authors have nothing to report.

## Conflicts of Interest

The authors declare no conflicts of interest.

## Data Availability

Raw data file included with submission. The raw data that support the findings of this study are available from the corresponding author upon request.
